# Incorporating Local Adaptation Into Species Distribution Modeling of *Paeonia mairei*, an Endemic Plant to China

**DOI:** 10.3389/fpls.2019.01717

**Published:** 2020-01-28

**Authors:** Qihang Chen, Yijia Yin, Rui Zhao, Yong Yang, Jaime A. Teixeira da Silva, Xiaonan Yu

**Affiliations:** ^1^ College of Landscape Architecture, Beijing Forestry University, Beijing, China; ^2^ Beijing Key Laboratory of Ornamental Plants Germplasm Innovation & Molecular Breeding, Beijing, China; ^3^ National Engineering Research Center for Floriculture, Beijing, China; ^4^ Beijing Laboratory of Urban and Rural Ecological Environment, Beijing, China; ^5^ Independent Researcher, Kagawa–ken, Japan

**Keywords:** climate change, endemic species, local adaption, MaxEnt, species distribution

## Abstract

*Paeonia* (Paeoniaceae), a culturally and economically important plant genus, has an isolated taxonomy while the evolution of this genus is unclear. A plant species endemic to southwest China, *Paeonia mairei* is precious germplasm for evolution-related research and cultivar improvement, and its conservation is urgent. However, little is known about its patterns of habitat distribution and responses to climate change. Using 98 occurrence sites and data of 19 bioclimatic variables, we conducted principal component analysis and hierarchical cluster analysis to delineate different climatic populations. Maximum entropy algorithm (MaxEnt) was applied to each population to evaluate the importance of environmental variables in shaping their distribution, and to identify distribution shifts under different climate change scenarios. We also applied MaxEnt to all of the *P. mairei* presence sites (P_Whole) to evaluate the need to construct separate species distribution models for separate populations rather than a common approach by treating them as a whole. Our results show that local adaptation exists within the distribution range of *P. mairei* and that all presence sites were clustered into a western population (P_West) and an eastern population (P_East). Two variables (precipitation of the driest month and temperature seasonality) are important when shaping the distribution of P_West, and another two variables (mean diurnal range and mean temperature of the wettest quarter) are important for P_East. Both populations are likely to shift upward under climate change, while P_East may lose most current suitable areas while P_West may not. P_Whole produced a narrower area compared to the combination of P_West and P_East but a suitable area (south Chongqing) may have been missed in the prediction. Accordingly, a population-based approach in constructing a species distribution model is needed to provide a detailed appreciation of the distribution of *P. mairei*, allowing for a population-based conservation strategy. In this case, it could include assisted migration to new and suitable distribution areas for P_West and *in situ* conservation in high elevation regions for P_East. The results of our study could be a useful reference for implementing the long-term conservation and further research of *P. mairei*.

## Introduction

Over the past 100 years, anthropogenic greenhouse gas emissions have increased and resulted in unequivocal global warming, increasing temperatures by approximately 1.0°C more than pre-industrial levels, which are likely to increase 3.2°C by 2100 if emissions continue to increase at the current rate (Hoegh-Guldberg et al., 2018). Besides raising mean temperature, particular regional climate will be characterized by warming due to extreme temperatures, frequent heavy precipitation, and/or intense drought (Hoegh-Guldberg et al., 2018), which significantly drive biodiversity loss, habitat fragmentation, and changes in the spatial patterns of plant species (Li et al., 2013; Costion et al., 2015; Warren et al., 2018). To face the challenge of climate change, predictions will be useful to alert researchers and policy-makers to potential future risks and can support the development of proactive strategies to reduce the impact of climate change on biodiversity and target species (Pereira et al., 2010).

*Paeonia*, the only genus in the Paeoniaceae, has an isolated taxonomy and ancient origin (Pan, 1995; Zhou et al., 2018). Consisting of 33 species, *Paeonia* is mainly distributed in the northern temperate zone, and China is reportedly its center of origin and diversity (Pan, 1995; Hong, 2011). Peonies have been used by humans for more than 3000 years, and they are now widely cultivated for medicinal, horticultural, and edible purposes (Wang, 2006). *Paeonia mairei* Lévl., assumed to originate from two ancient populations (Zhou et al., 2018), is an endemic species to southwestern China (Pan, 1995). This germplasm is important for further studies on the evolution of the genus *Paeonia* and for breeding research for medicinal and horticultural industries. However, *P. mairei* is endangered with a shrinking distribution range (Yang et al., 2017). As demonstrated by our field survey from 2017-2019 in southwest China (**Supplementary File 1**), most populations show a limited number of individuals, while it is difficult to find individuals in some documented distribution regions, such as Nanchuan and Zhaojue counties.

Geographic distribution and response pattern to climate change are unclear for peony species, so developing a scientifically-sound conservation strategy under climate change will be difficult. As a common tool to achieve this objective, species distribution models (SDMs) typically correlate the presence (or absence) of a species at multiple locations with relevant environmental covariates to estimate habitat preferences or to predict their distributions (Hijmans and Graham, 2006; Kearney et al., 2010; Gomes et al., 2018). However, the effect of local adaptation is largely ignored in most SDMs studies in which all populations of a species were assumed to respond consistently to the environmental conditions experienced by the entire species. Considering that broad climate tolerance at the species level is generally comprised of narrower, locally adapted tolerance at the scale of populations (Atkins and Travis, 2010; Peterson et al., 2019), failure to account for local adaptation, as common SDM-based studies do, may introduce errors into forecasts about geographic distributions and the future viability of a species as a whole (Pearman et al., 2010), and precludes analysis of the fates of specific intraspecific lineages or the maintenance of genetic variation in ecologically important traits (D'Amen et al., 2013; Marcer et al., 2016). Several attempts have recently been made to incorporate local adaptation into ecological analyses under climate change (Pearman et al., 2010; Hallfors et al., 2016; Peterson et al., 2019), which indicates that there can be an effect of discriminating populations in response to climate change. As Hallfors et al. (2016) proposed, local adaptation probably exists within species that inhabit separate climatic environments across their range, and such cases include species with spatially distinct populations, species with taxonomic confusion, as well as subspecies or endangered species that are discontinuous across their range. Until further experiments are able to elucidate the existence of local adaptation, an approach to model uncertain populations separately—in addition to whole-species modeling—is needed to attain more comprehensive information for conservation strategies.

As an endangered species endemic to southwestern China, *P. mairei* is mainly located in two mountain systems, i.e. Hengduan mountain in middle Sichuan and Qinling Mountains in middle Shaanxi. By considering the spatial distinction between these two mountain systems, in this study, we assumed the existence of local adaptation between populations of *P. mairei* in both mountain systems. As a result, we divided all recorded presence sites of *P. mairei* into two populations based on two separate distribution ranges, including the western population (P_West) with presence sites in Hengduan mountain and the eastern population (P_East) with presence sites in Qinling Mountains. With two separate populations, we applied multivariate statistical analysis to verify whether each population experienced different climatic conditions, and then used the maximum entropy algorithm (MaxEnt) to simulate the migration trend of the potential distribution of each population under several climate change scenarios. The aims of the present study were to: i) identify probable specific populations with different local adaptations; ii) identify the key climate variables that shape the distribution range of each population; and iii) project the change of habitat distribution for each population under global climate change. These results will contribute to a better understanding of the environmental demands of *P. mairei* and provide a theoretical basis and guidance for conservation strategies for this rare Chinese endemic germplasm.

## Material and Methods

### Species Occurrence Data

*P. mairei* is a herbaceous perennial with thick roots that is often found in deciduous broad-leaved forests, where it is moist and shaded (Hong, 2011). Native records of the occurrence of *P. mairei* were collected from three sources: (1) field surveys during 2016 and 2018 in southwest China, (2) published literature (Hong, 2010; Jian et al., 2010; Zhang et al., 2016; Yang et al., 2017), and (3) databases including the Global Biodiversity Information Facility (GBIF, http://www.gbif.org), the National Specimen Information Infrastructure (NSII, http://www.nsii.org.cn/), and the Chinese Virtual Herbarium (CVH, http://www.cvh.org.cn/) databases. Originally, 98 presence records with exact coordinates were obtained. These were spatially filtered with the help of ArcGIS Pro (version 2.4.2, Esri, Redlands, CA, USA), so that only one record occurred within each grid cell (20 × 20 km) to correct sampling bias.

### Study Region

**Figure 1:**
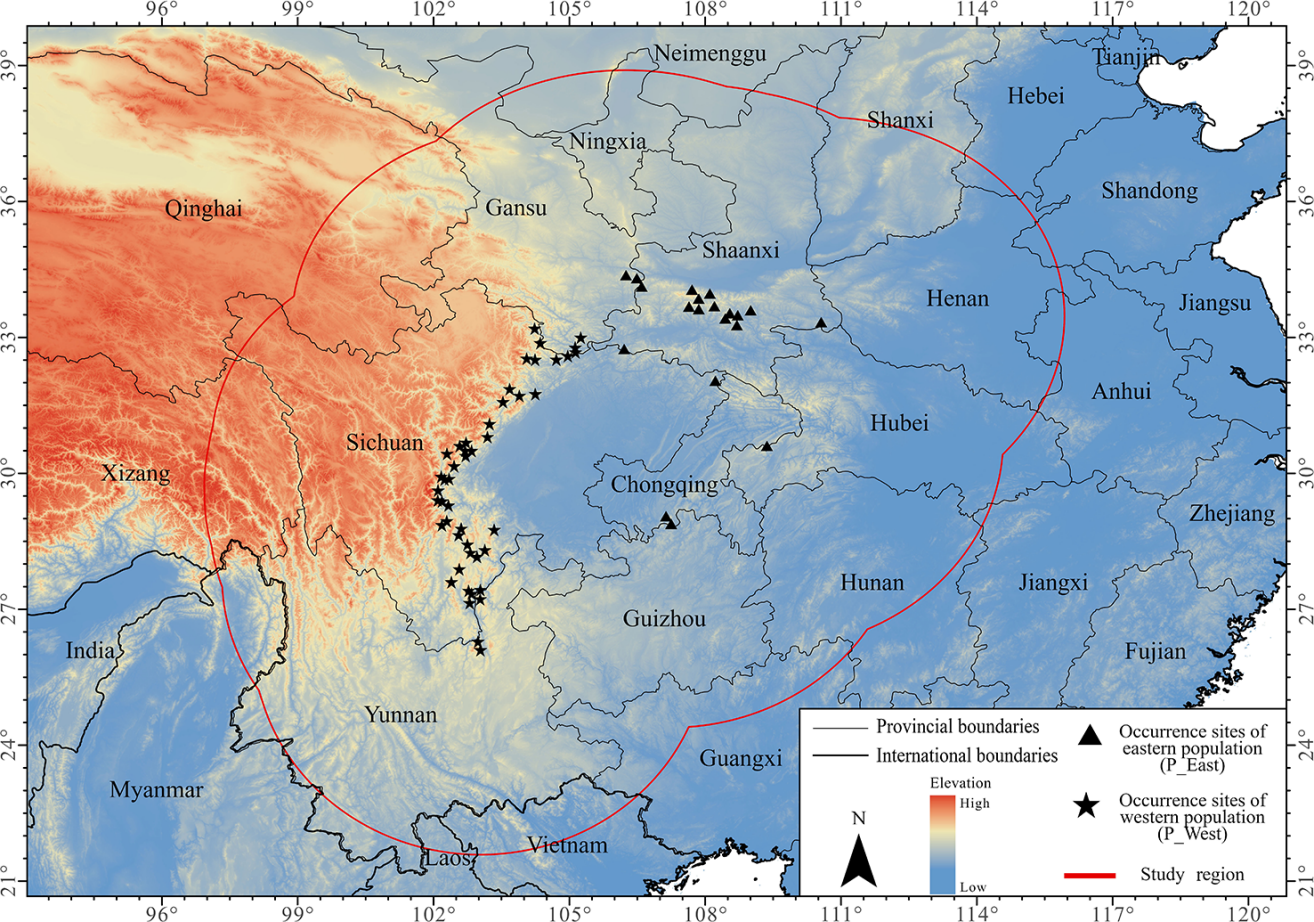
Occurrence sites of *Paeonia mairei*. All occurrence sites were divided into two populations based on geographical locations: sites in Hengdaun Mountains were referred to as western population (P_West) and sites in Qinling Mountains were referred to as eastern population (P_East).

In this study, we established a study region using a 500-km buffer around the occurrence points of *P. mairei* (**Figure 1**). The chosen study region included all occurrences of the species, minus extrapolation when projecting under other climate conditions or larger dominants (Hallfors et al., 2016). When taking anthropogenic-assisted migration into consideration for the further conservation of *P. mairei*, an ideal study region should cover areas not only where it can disperse biologically in the near future, but also where it might migrate under longer periods of climate change and possible candidate sites for assisted migration. As a consequence, we used the buffer obtained from the whole *P. mairei* species for distribution modeling of each population.

### Climate Data

Initially, 19 bioclimatic variables that reflected the *P. mairei* distribution data collection (mean value from 1960 to 1990) with a general spatial resolution of 30 seconds from the WorldClim dataset (http://www.worldclim.org) (Hijmans et al., 2005), were selected to model the current distribution of *P. mairei*. The degree of conﬁdence of future climate-change projections depends on global climate model (GCM) performance, but no single climate model is superior to forecast climatic features, so we assumed an averaged multi-model ensemble climate forecast for our projection in future scenarios. We downloaded future climate data of three GCMs (Beijing Climate Center Climate System Model version 1.1, BCC-CSM1.1; the Community Climate System Model version 4 CCSM4; and an earth system model based on the Model for Interdisciplinary Research on Climate, MIROC-ESM) from the WorldClim dataset, which was statistically downscaled from climate models for the fifth report of the International Panel for Climate Change (IPCC, 2014). Equally-weighted mean values of three GCMs were calculated to obtain a suite of future climate data including 19 bioclimate variables under three representative concentration pathways (RCPs): RCP 2.6, RCP 6.0, and RCP 8.5. RCPs are used to project future climate situations based on human economic activity, land use patterns, climate policy, and other factors. They include a stringent mitigation scenario (RCP 2.6), two intermediate scenarios (RCP 4.5 and RCP 6.0), and one scenario with very high greenhouse gas emissions (RCP 8.5) (IPCC, 2014). Under each RCP, we used climate data of two periods (2050s and 2070s; averaged for 2041–2060 and 2061–2080) in the 21st century to project habitat distribution changes.

Highly correlated variables will mislead the importance of variables and response curves in further research, so we performed a variance inflation factors (VIF) analysis to help eliminate highly correlated variables (Merow et al., 2013). For the analysis, we extracted all values of 19 climate variables from a sample of 10,000 locations within the study region. As a consequence, we retained six variables with VIF values less than 10 for further research even though a threshold of five (Suwal et al., 2018) or three (Di Febbraro et al., 2016) have been suggested, considering that machine learning methods such as MaxEnt can cope with some degree of collinearity (Elith et al., 2011). With the help of ArcGIS Pro, all environmental variables were preprocessed in American Standard Code for Information Interchange (ASCII) format with a general spatial resolution of 30 seconds (also referred to as 1 km spatial resolution).

### Population Grouping

As geographic distinction often indicates climatic adaptation (Hallfors et al., 2016), we divided all occurrence sites into western (P_West) and eastern (P_East) populations (**Figure 1**), which are located in two different mountain systems (Qinling Mountains and Hengduan Mountains, respectively). Principal component analysis (PCA) (Hallfors et al., 2016), hierarchical cluster analysis (HCA), and linear discriminant analysis (LDA) were then used to check whether different climate conditions exist between two populations, and PCA was conducted with all 19 variables using the “prcomp” function in the “stats” package of R software (version 3.5.3). We used the first two principal components and a 95% confidence interval during clustering to define the populations with the help of the “ggplot2” package (Wickham, 2016) in R. LDA and HCA were conducted with six variables chosen through the VIF approach, and with the help of the “cluster” (Maechler et al., 2019) and “MASS” (Venables and Ripley, 2002) package in R, we selected the method for HCA with the highest agglomerative coefficient value. We built the linear discriminant function by randomly selecting 50% of all presence sites, and tested the function with this 50% of sites. This process was repeated 1000 times and the mean misjudgment rate was calculated to check differences in climatic adaptation between the two populations. In addition to the divided populations, SDMs were constructed using all present sites together (P_Whole), which is a common practice when conducting SDM-based research. In other words, we constructed SDMs for *P. mairei* using two approaches, one that produced separate SDMs for separate populations (P_West and P_East) and another that produced a single SDM for the entire species and that encompassed both populations (P_Whole).

### Constructing SDMs

With the help of the “Biomod2” (Thuiller et al., 2019) package in R, we selected MaxEnt for our species distribution modeling through a pre-test, in which we constructed SDMs using eight common algorithms (Generalized Linear Model, GLM; Generalized Boosting Model or usually called Boosted Regression Trees, GBM; Classification Tree Analysis, CTA; Artificial Neural Network, ANN; Surface Range Envelope, SRE; Multiple Adaptive Regression Splines, MARS; Random Forest, RF and Maximum Entropy, MaxEnt) with P_Whole. All algorithms were repeated 10 times with 50% of presence sites randomly selected for testing and three common evaluating statistics (Cohen's Kappa, KAPPA; True Skill Statistic, TSS and Areas Under the Receiver Operating Characteristic Curve, AUC) were selected to evaluate the performance of SDMs (**Supplementary File 2**). MaxEnt (Phillips et al., 2006) is a commonly used SDM algorithm for presence-only data, and it has also been shown to perform well in comparison to different algorithms (Reside et al., 2019) although explicit relationships between suitability and environmental variables are difficult to obtain from such a machine-learning algorithm (Phillips et al., 2006). We used MaxEnt (version 3.4.1) to constructed SDMs for three suites of presence data (P_West, P_East, and P_Whole) in the current situation and in two future periods under four RCPs, as explained in section *Climate Data*. With the help of the ENMeval package in R, we evaluated the performance of models with regulation multiplier values ranging from 0 to 4 (increments of 0.5) and with six different feature class combinations (L, LQ, H, LQH, LQHP, LQHPT; where L = linear, Q = quadratic, H = hinge, P = product, and T = threshold). From those settings (regulation multiplier and feature class combination) with the top 10% of average test areas under the receiver operating characteristics curve (AUC) values, the one with the lowest value of the Akaike information criterion corrected for small samples sizes (AICc) was selected as the best for constructing SDMs. Random 50% of the occurrence data was used for modeling, the remaining 50% for testing, and 10 times bootstrap to obtain 10 SDMs for each data suite in each scene. Within MaxEnt software (version 3.4.1), threshold-independent receiver-operating characteristic (ROC) analyses were conducted, and the areas under the receiver operating characteristics curve (AUC) were calculated to check the performance of SDMs (Phillips et al., 2006). AUC, ranging from 0 to 1, is one of the most popular parameters to evaluate the performance of an SDM, and any SDM with a test AUC value under 0.85 was removed to ensure the good performance of SDMs for further analysis. For each remaining SDM, the continuous probability of habitat suitability was converted to binary outputs of suitable or unsuitable areas with the help of maximum sensitivity plus specificity threshold, which can minimize the mean of the error rate and has been widely used in SDMs (Liu et al., 2013). Several binary maps, which were obtained for each data suite in each scene, were assembled to create a final suitable-unsuitable map using a majority vote approach (Hallfors et al., 2016), i.e., a location was considered to be suitable if more than 60% of models projected that cell to be suitable.

To understand which climate variables may be important and differ among populations (or species), we used the permutation importance and jackknife measures (Shcheglovitova and Anderson, 2013) in MaxEnt to assess the relative contribution of each environmental variable, to identify the most important variables and determine the predicted distribution of the modeled entity. Response curves of all variables in the models were identified and examined to check the response of each population (or species) to different variable values.

### Geospatial Analysis

We obtained three suite results for three suites of presence data (P_West, P_East, and P_Whole), which allowed us to determine the current distribution and changes in the future separately. To create a summary description of the predicted distribution change under different climate scenarios for each population, we calculated the area and centroid of suitable distribution, and comparisons were made using different scenarios and periods. To further display suitability changes, projections under the same RCP were compared with the current distribution and ensembled to a discrete map to illustrate changes of suitability in each cell, resulting in eight kinds of situations that could occur in different cells. Simultaneously, we combined the binary results of P_West and P_East to obtain a new suite of binary results named P_Combination, in which a cell was considered suitable for *P. mairei* if it was suitable for both P_West and P_East. We compared the distribution of P_West with that of P_East, and P_Combination with P_Whole, to assess niche overlap between them and check the need to conduct separate SDMs rather than entire SDMs. Schoener's D values (Sch_D) (Schoener, 1968) and corrected modified Hellinger distance (Cor_I) (Warren et al., 2008) were calculated to measure niche overlap between SDMs. Both statistics ranged from 0 (species with completely discordant niches) to 1 (species with identical niches). All calculations were performed in R with the help of packages including: “sp” (Pebesma and Bivand, 2005), “raster” (Hijmans, 2019), “rgeos” (Bivand and Rundel, 2019), and “dismo” (Hijmans et al., 2017), while ArcGis Pro was used for plotting all geographic distribution maps.

## Results

### Population Grouping

**Figure 2:**
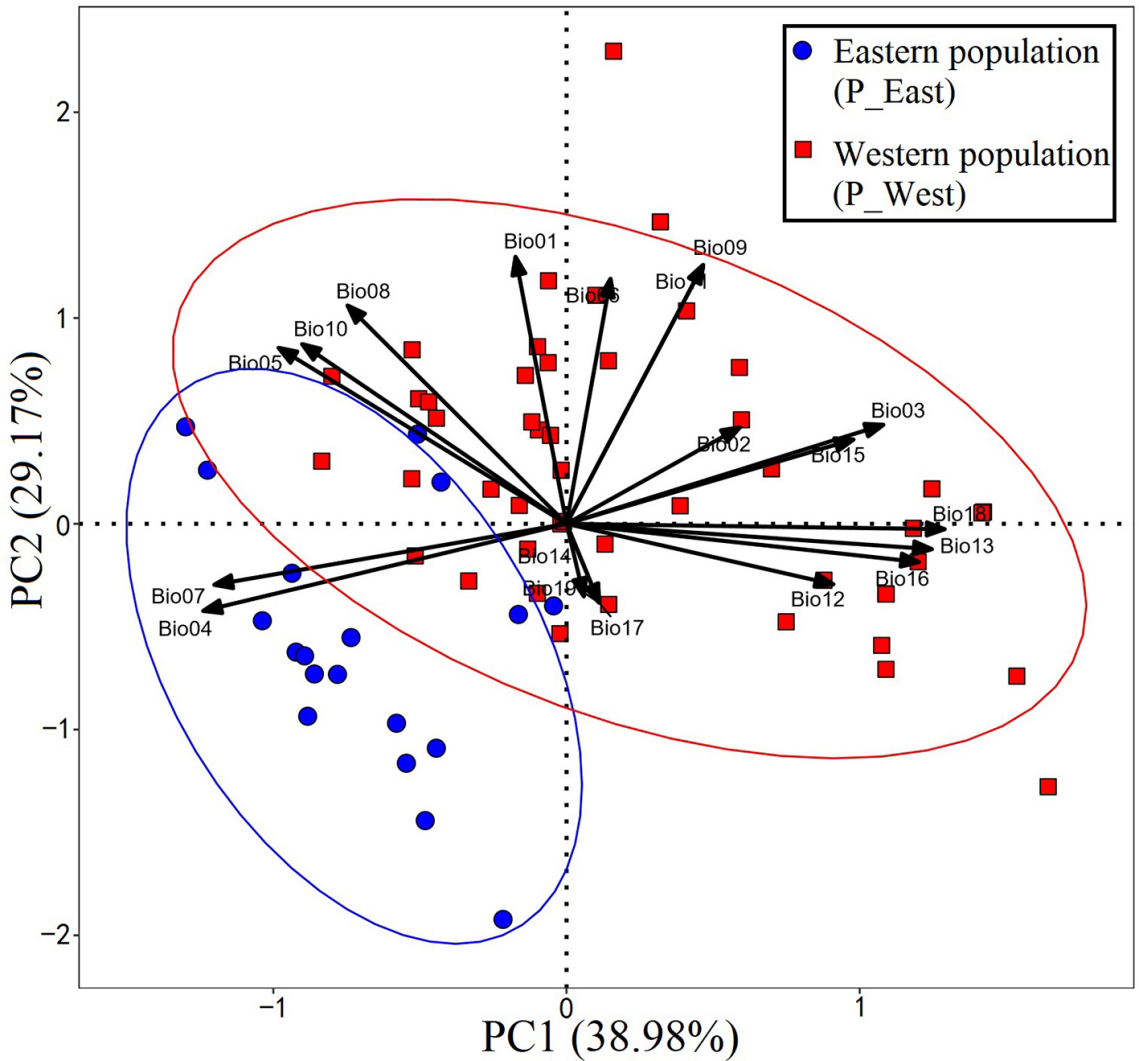
Result of principal component analysis. P_East occupies a niche featured by high Bio04 (temperature seasonality) and Bio07 (annual temperature range), while P_West features high Bio09 (mean temperature of the driest quarter) and Bio11 (mean temperature of the coldest quarter).

**Figure 3:**
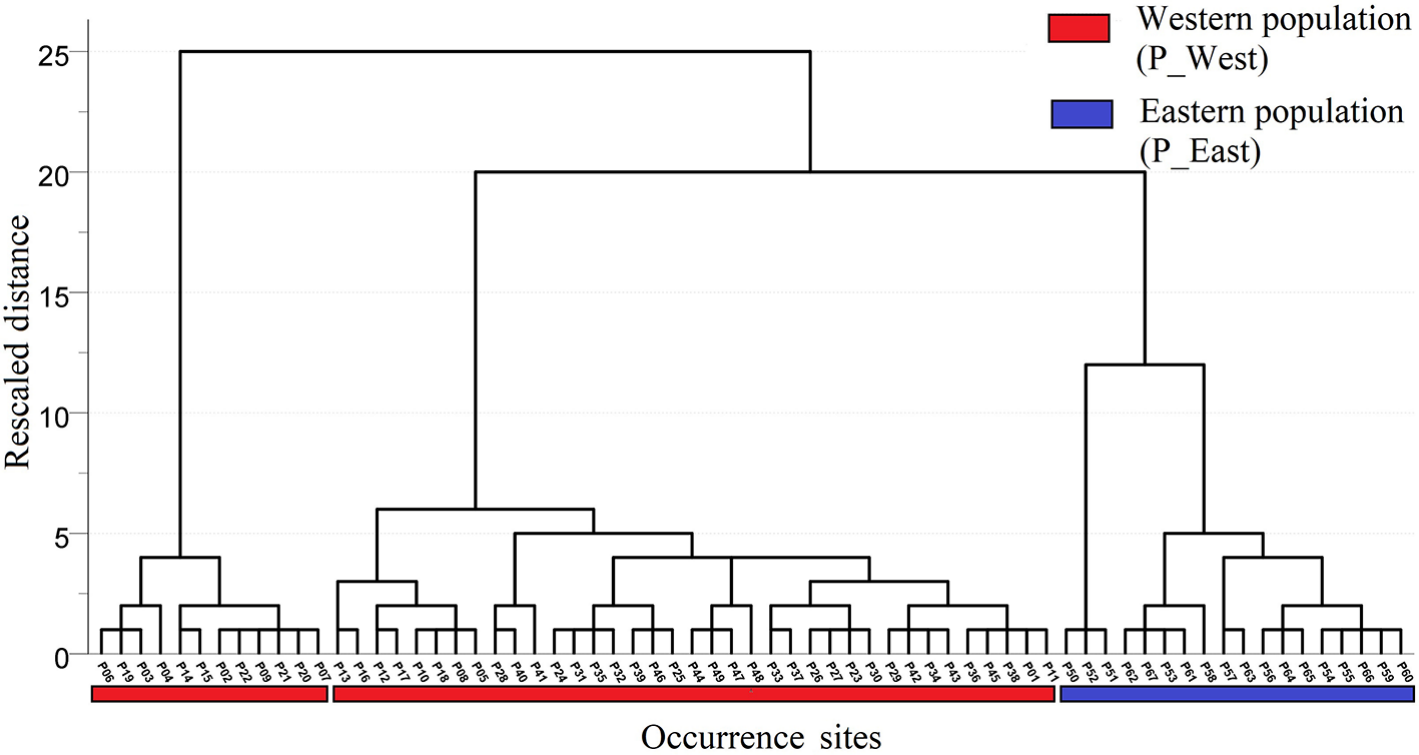
Result of hierarchical cluster analysis. All sites of occurrence could be grouped into three groups: two groups for P_West and one group for P_East.

A total of 67 presence records (**Figure 1**) with geo-coordinates were obtained for further analysis through spatial filtering. Three principal components, which were obtained through PCA based on 19 variables, explained 93.39% of cumulative variance (**Table 1**). The distribution of 67 presence sites was drawn based on the first two principal components, which explained 68.15% of all variance. Despite some overlap between the two populations, P_West and P_East were located separately in PCA maps: P_East occupied a niche featured by high Bio04 (temperature seasonality) and Bio07 (annual temperature range), while P_West featured high Bio09 (mean temperature of the driest quarter) and Bio11 (mean temperature of the coldest quarter) (**Figure 2**). The HCA result (**Figure 3**) shows that all sites of occurrence can be grouped into three groups: two groups for P_West and one group for P_East. We obtained a mean misjudgment rate of 1.67% with a standard deviation of 2.1% from LDA, which justified the difference between P_West and P_East. The combined results from HCA and PCA indicate that divergence in climate adaptation exists between the two populations, despite little overlap and divergence existing within P_West. Considering that a limited number of presence sites in further clustering may not support a good performance of model training and testing, we finally selected P_West and P_East for our further research.

**Table 1:** Correlation between components and variables and variance explained.

		Bioclimatic variables	PC 1	PC 2	PC 3
Loadings	Bio01	Annual mean air temperature	−0.128	0.951	0.279
	Bio02	Mean diurnal temperature range	0.433	0.344	−0.646
	Bio03	Isothermality	0.792	0.353	−0.409
	Bio04	Temperature seasonality	−0.908	−0.31	0.151
	Bio05	Max temperature of the warmest month	−0.72	0.627	0.239
	Bio06	Min temperature of the coldest month	0.11	0.873	0.428
	Bio07	Annual temperature range	−0.88	−0.217	−0.18
	Bio08	Mean temperature of the wettest quarter	−0.547	0.777	0.265
	Bio09	Mean temperature of the driest quarter	0.341	0.922	0.165
	Bio10	Mean temperature of the warmest quarter	−0.661	0.64	0.346
	Bio11	Mean temperature of the coldest quarter	0.341	0.922	0.165
	Bio12	Annual precipitation	0.667	−0.215	0.677
	Bio13	Precipitation of the wettest month	0.913	−0.09	0.24
	Bio14	Precipitation of the driest month	0.043	−0.259	0.93
	Bio15	Precipitation seasonality	0.717	0.3	−0.555
	Bio16	Precipitation of wettest quarter	0.881	−0.136	0.329
	Bio17	Precipitation of driest quarter	0.081	−0.28	0.929
	Bio18	Precipitation of the warmest quarter	0.945	−0.019	0.265
	Bio19	Precipitation of the coldest quarter	0.081	−0.28	0.929
Eigenvalues			7.406	5.543	4.795
Percent of variance			38.977	29.174	25.239
Cumulative percent of variance			38.977	68.151	93.39

### Current Distribution of Suitable Habitat

**Figure 4:**
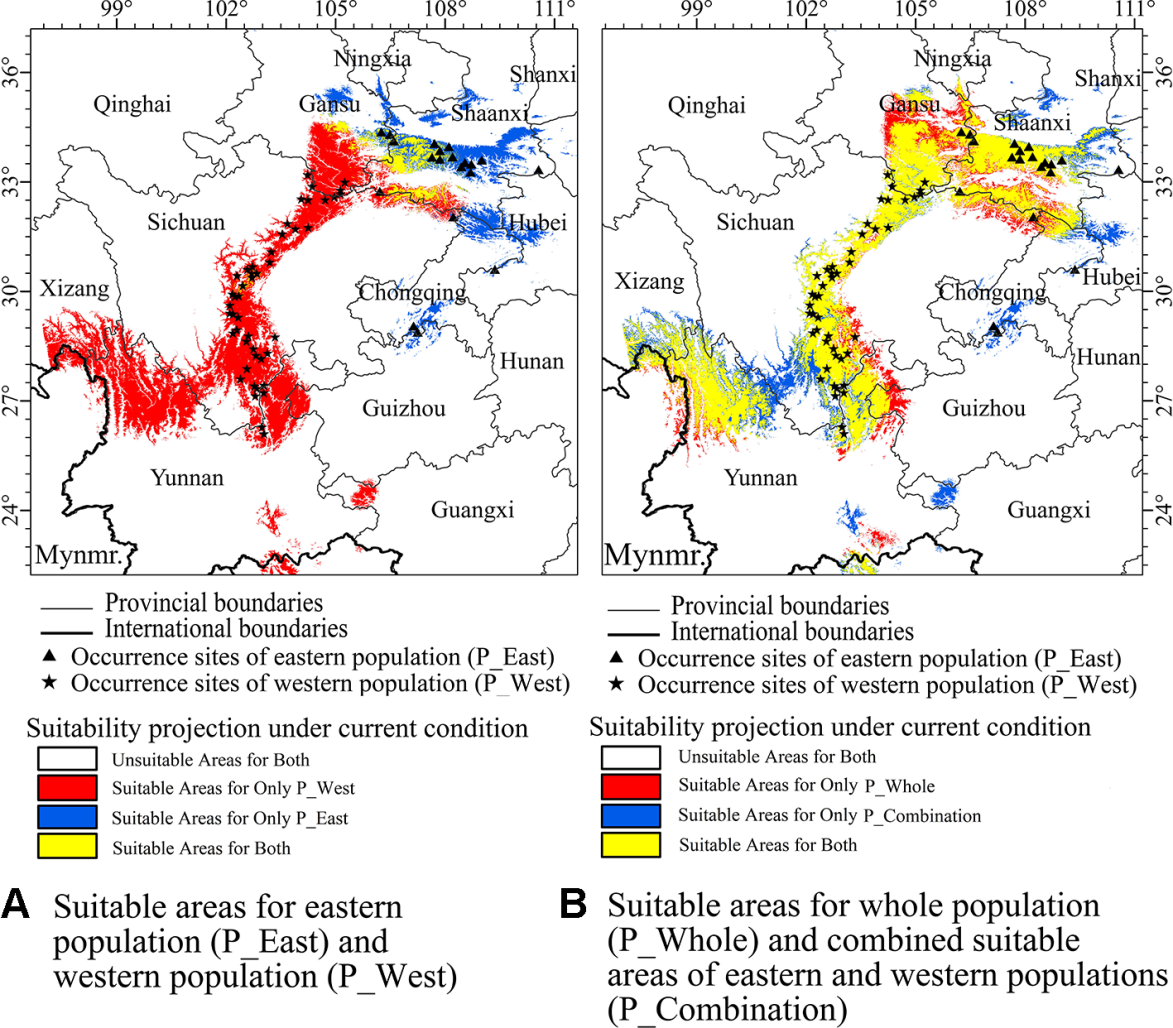
Currently suitable areas which were projected by maximum entropy algorithm (MaxEnt) for western population (P_West), eastern population (P_East), whole population (P_Whole), and an combined result (P_Combination) from suitable areas of P_West and P_East, in which a cell was considered suitable for *P. mairei* if it was suitable for P_West or P_East. **(A)** P_West was predicted to occur in middle Sichuan, south Gansu, north Yunnan, and a part of south Shaanxi. P_East was predicted to mainly occur in middle Shaanxi. **(B)** Compared with the results of P_West and P_East, P_Whole lost a suitable region in south Chongqing, which was reported to be suitable for *P. mairei*.

Based on three evaluated metrics from ENMeval (**Supplementary File 3**), a regulation multiplier of 0.5 and a feature class combination of linear and quadratic (LQ) was selected for all SDMs of three suites of data. After removing SDMs with a test AUC under 0.85, mean testing AUCs of SDMs using three suites of presence data ranged from 0.937 to 0.980 (**Supplementary File 4**), suggesting that most models performed well and generated excellent evaluations (Phillips et al., 2006). Current suitable areas for P_West were predicted to exist in middle Sichuan, south Gansu, north Yunnan, and part of south Shaanxi, while regions along the eastern side of Hengduan Mountains were suitable for P_West. Current suitable areas for P_East were predicted to mainly occur in middle Shaanxi, as well as regions in west Hubei and south Chongqing, which are located in the Qinling, Daba, and Dalou Mountains (**Figure 4A**). The western region of Daba Mountains was predicted to be suitable for P_West, while most of this area was predicted to be unsuitable for P_East. It seems inconsistent with our assumption that P_West is suitable in Hengduan Mountains and P_East is suitable in Qinling and Daba Mountains. Current suitable areas for P_Whole were predicted to mainly occur in south Gansu, middle Shaanxi, middle Sichuan, and north Yunnan. After combining the three result maps, compared with the results of P_West and P_East, P_Whole lost a suitable region in south Chongqing, where the presence of *P. mairei* has been documented (**Figure 4**).

### Core Distribution and Area of Suitable Habitats

**Figure 5:**
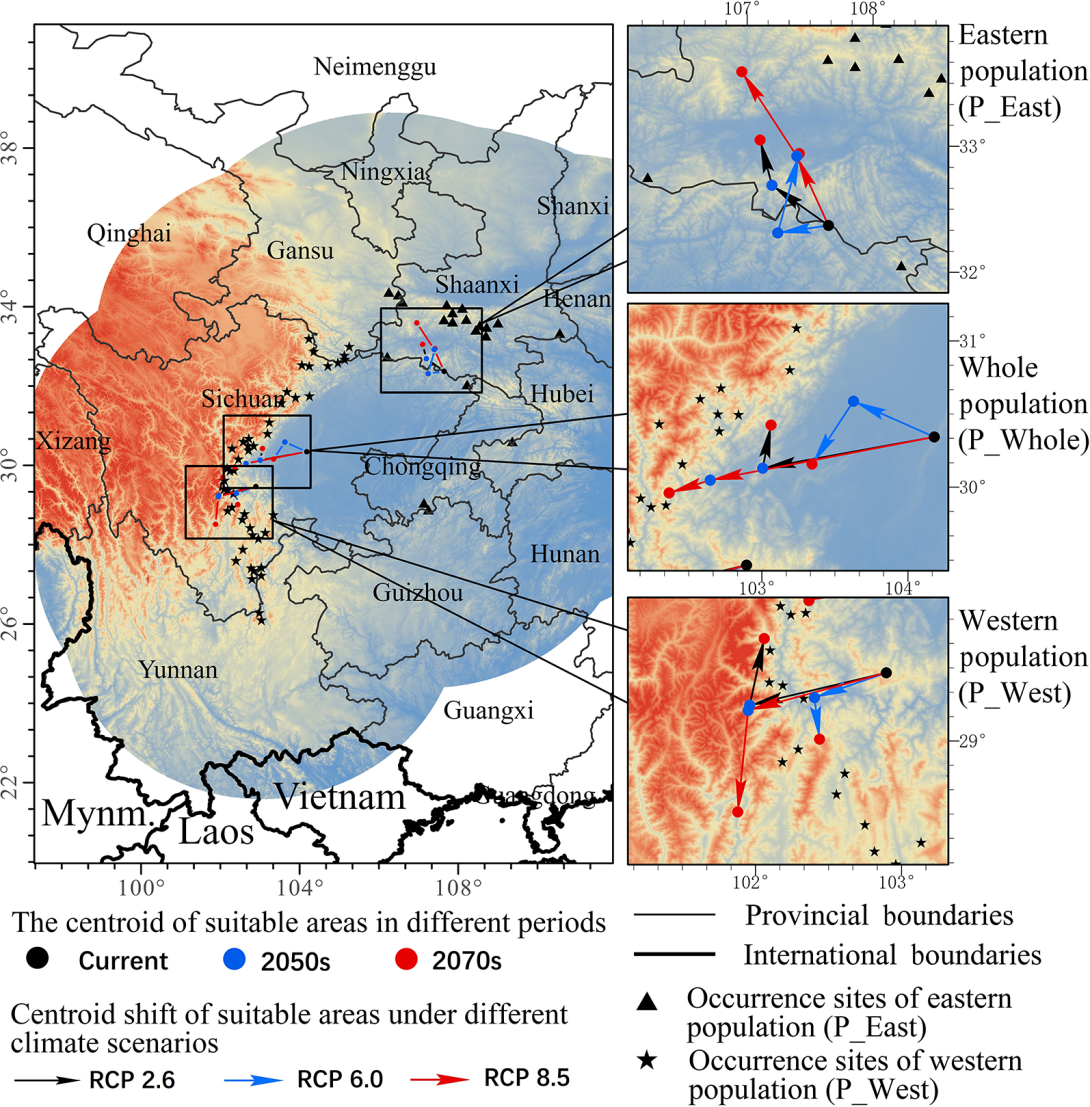
Core distributions of different species distribution models (constructed with eastern population (P_East), western population (P_West), and whole population (P_Whole) of *P. mairei*) and their changes under climate change, including two periods (from now until the 2050s and from the 2050s–2070s) under three climate scenarios (RCP2.6, RCP6.0, and RCP8.5). P_East shows a north shift trend while P_West and P_Whole may shift west, although the overall trend of all populations was northwards.

To grasp an overall understanding of distribution shifts, the centroid of each SDM was calculated, and vectors were drawn to illustrate the direction and distance of centroid shifts of both populations under different climatic scenarios (**Figure 5**). The centroid of the current habitat of P_West was predicted to be located in the middle of Sichuan province (102.90 E and 29.47 N), and might shift west in the 2050s in RCP 2.6 (101.96 E and 29.24 N) and RCP 8.5 (101.95 E and 29.21 N). The centroid of suitable area might shift north in the 2070s in RCP 2.6 (102.06 E and 29.7 N), but might shift south in the 2070s in RCP 8.5 (101.88 E and 28.51 N). Overall, the core distribution of P_West showed a westward-shifting trend under the three pathways since western regions have a higher altitude. The centroid of the current habitat of P_East was predicted to be located in south Shaanxi province (107.65 E and 32.37 N). It might shift north in the 2050s in RCP 2.6 (107.2 E and 32.69 N) and RCP 8.5 (107.39 E and 32.92 N) and might shift further north in the 2070s in RCP 2.6 (107.1 E and 33.05 N) and RCP 8.5 (106.96 E and 33.59 N). Overall, the core distribution of P_East in different pathways all showed a north-bound shift since northern areas are also characterized by higher altitudes. The centroid of the current habitat of P_Whole, which was predicted to be located in middle Sichuan province (104.18 E and 30.34 N), and which is near the core distribution of P_West, presented similar shifting trends. It might shift west in the 2050s in RCP 2.6 (103.01 E and 30.13 N) and RCP 8.5 (102.65 E and 30.05 N), and in the 2070s in RCP 2.6 (103.06 E and 30.42 N) and RCP 8.5 (102.37 E and 29.96 N). Overall, the core distribution of the three suites of SDMs showed an upward-shifting trend under the three RCPs.

**Figure 6:**
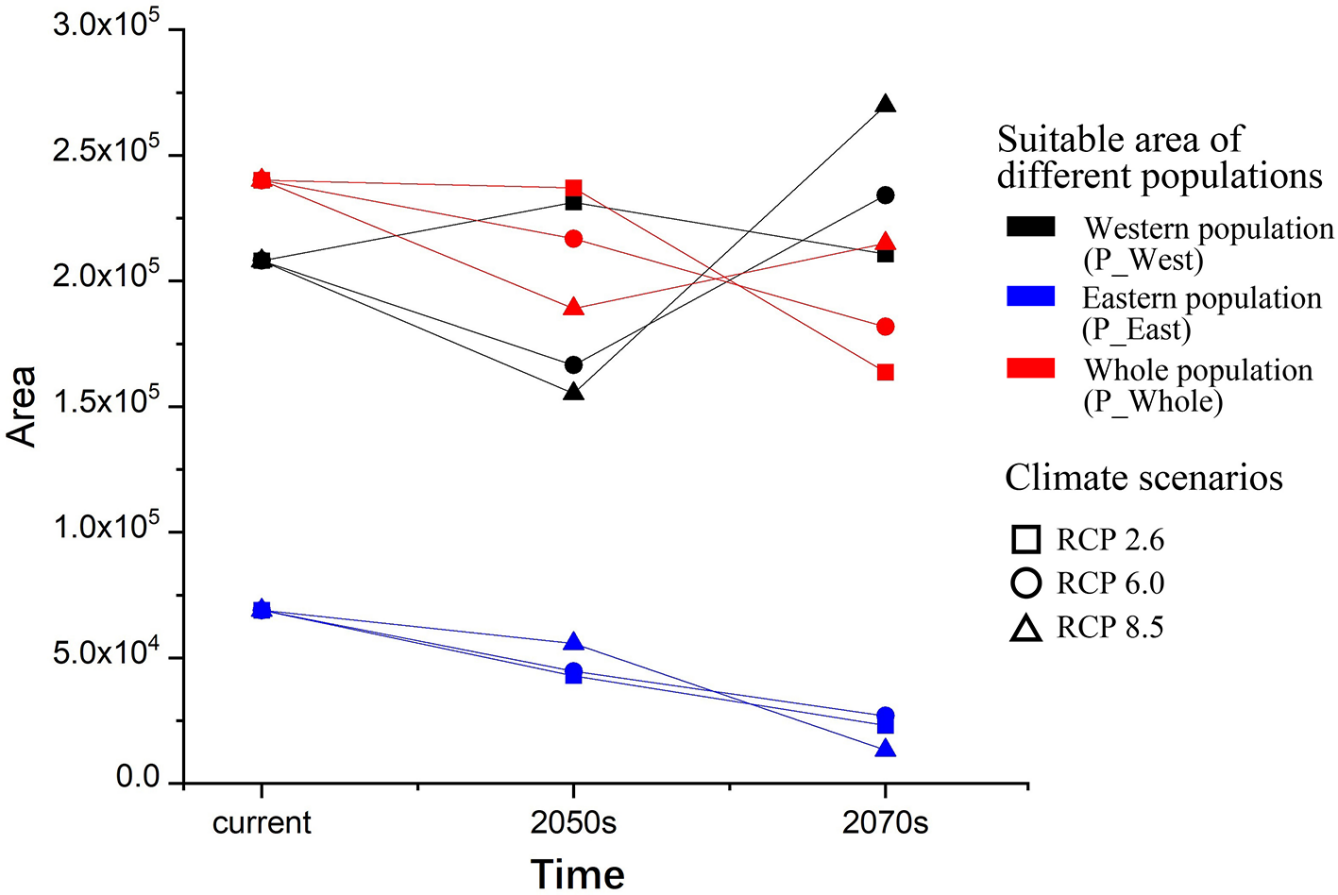
Suitable areas and their changes under climate change. Both areas (P_East and P_Whole) may decrease under climate change in the 2070s under three RCPs, while P_West may increase slightly in the 2070s under three RCPs although it may face a decrease in the 2050s under RCP6.0 and RCP8.5.

The current suitable area of P_West was predicted to be 2.08 × 10^5^ km^2^, and it might increase in the 2050s in RCP 2.6 (2.31 × 10^5^ km^2^) but decrease in RCP 8.5 (1.55 × 10^5^ km^2^), and might also increase in the 2070s in RCP 2.6 (2.11×10^5^ km^2^) and RCP 8.5 (2.7 × 10^5^ km^2^) (**Figure 6**). The current suitable area of P_East was predicted to be 6.90 × 10^4^ km^2^, and it might decrease in the 2050s in RCP 2.6 (4.28×10^4^ km^2^) and RCP 8.5 (5.58×10^4^ km^2^) and might keep decreasing in the 2070s in RCP 2.6 (2.31×10^4^ km^2^) and RCP 8.5 (1.32×10^4^ km^2^). The current suitable area of P_Whole was predicted to be 2.40×10^5^ km^2^, and it might decrease in the 2050s in RCP 2.6 (2.37×10^5^ km^2^) and RCP 8.5 (1.89×10^5^ km^2^), and might keep decreasing in the 2070s in RCP 2.6 (1.64×10^5^ km^2^), and might increase slightly in RCP 8.5 (2.15×10^5^ km^2^). Overall, the suitable area of P_East might decrease sharply under climate change, P_Whole might decrease slightly while P_West might remain unchanged.

### Changes of Suitable Habitat

**Figure 7:**
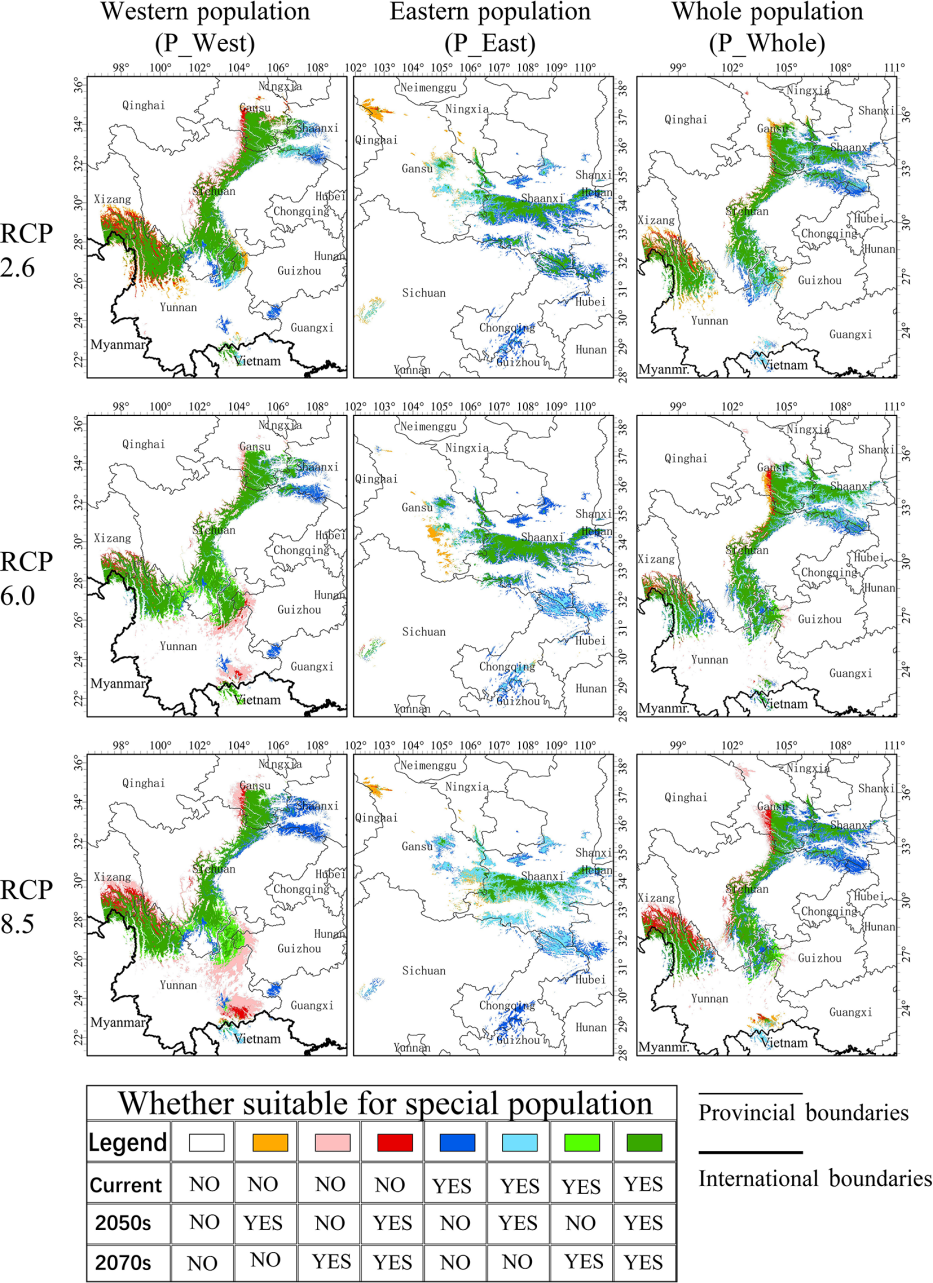
Changes to suitable areas under climate change. Eight colors refer to eight situations that occurred in specific locations. Green or grass green refer to a relatively stable region that is suitable for *P. mairei*; blue or sky blue refer to a threatened region where it will no longer become suitable from the 2050s or 2070s; red or pink refer to a promising region where conditions will become suitable from an unsuitable state from the 2050s or 2070s.

Most current suitable areas of P_West were predicted to remain suitable under the three RCPs, including south Gansu, middle Sichuan, and northwest Yunnan, but a large part of south Sichuan and north Yunnan might face a loss of suitability in the 2050s and recovery in the 2070s in RCP 8.5 (**Figure 7**). The predicted increase in suitable areas of P_West in RCP 2.6 are mainly located on the western side of current suitable areas in Sichuan, and on the northern side of current suitable areas in Yunnan. Besides the two former regions, eastern Yunnan was also predicted to become suitable for P_West in RCP 8.5. The loss of suitable areas of P_West might occur primarily in south Shaanxi, on the eastern side of middle Sichuan, and in a part of western Guangxi. Most current suitable areas of P_East might become unsuitable in the three RCPs but maintain suitable areas in middle Shaanxi, so the overall area might decrease with a rise in RCP. Loss of suitable areas of P_East were predicted to include lowland in middle Shaanxi, and regions in south Gansu and south Chongqing. Middle Gansu might become suitable for P_East in the 2050s but unsuitable in the 2070s in the three RCPs. The situation experienced by P_Whole is predicted to be similar to that in P_West, where most current suitable areas might maintain their suitability under climate change, including middle Shaanxi, south Gansu, middle Sichuan, and northeast Yunnan. There might be an increase in suitable areas of P_Whole located to the west of current suitable areas in Sichuan and to the north of current suitable areas in Yunnan. In contrast, suitable areas predicted to be lost were mainly located in north Shaanxi and east of the current suitable area in middle Sichuan.

### Important Variables

**Figure 8:**
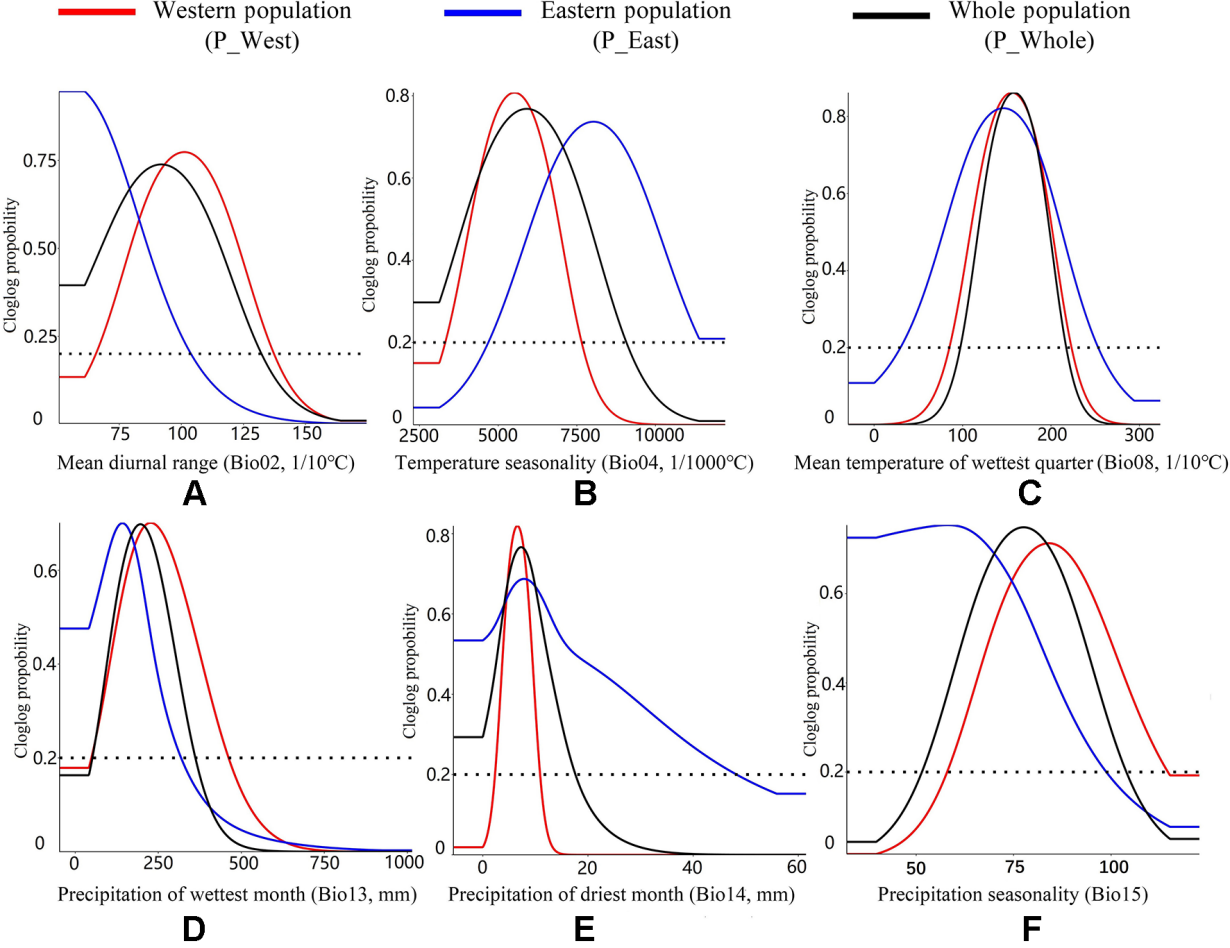
Response curves of all climate variables. The Y-axis shows the relative probability of the presence of *P. mairei* under a given value of each climate variable applied in our research. **(A)** Western population might adapt to a higher mean diurnal temperature range; **(B)** Eastern population might adapt to a higher temperature seasonality; **(C)** Two populations performs an stability in Mean temperature of wettest quarter; **(D)** Western population might adapt to a higher precipitation of wettest month; **(E)** Eastern population might adapt to a higher precipitation of driest of month; **(F)** Western population might adapt a higher precipitation seasonality.

For P_West, among the six climate variables adopted in the model, precipitation of the driest month (Bio14, 52.61%) (**Table 2**) and temperature seasonality (Bio4, 24.89%) made substantial contributions to the distribution model relative to other variables, indicating that these factors play important roles in its distribution. Mean diurnal range (Bio2, 11.89%) also made a substantive contribution to the distribution model, and the cumulative contribution of these three variables was 89.39%. Thresholds (presence probability > 0.2) for each variable were obtained through separate response curves: temperature seasonality (Bio4) ranged from 3.33 to 7.64°C, precipitation of the driest month (Bio14) ranged from 2.32 to 10.77 mm, and mean diurnal range (Bio2) ranged from 6.57 to 13.72°C (**Figure 8**).

**Table 2:** The permutation importance of the variables included in the MaxEnt models for *Paeonia mairei*.

The permutation importance (%)	Western populations (P_West)		Eastern populations (P_East)		All populations (P_Whole)	
	Average	SD	Average	SD	Average	SD
Mean diurnal range (Bio02)	11.89	5.81	**55.79**	18.11	**20.99**	7.52
Temperature seasonality (Bio04)	**24.89**	4.9	2.87	2.95	10.33	5.56
Mean temperature of wettest quarter (Bio08)	3.79	2.26	**16.17**	6.32	13.78	6.69
Precipitation of wettest month (Bio13)	1.81	1.76	3.82	6.45	6.08	3.69
Precipitation of driest month (Bio14)	**52.61**	5.76	12.43	12.2	**39.25**	9.31
Precipitation seasonality (Bio15)	5.02	2.76	8.91	11.97	9.57	4.31

Bold fonts refers to the contribution of the first and second important variables for each population.

For P_East, mean diurnal range (Bio2, 55.79%) made the greatest contribution to the distribution model, while mean temperature of the wettest quarter (Bio8, 16.17%) and precipitation of the driest month (Bio14, 12.43%) also made substantive contributions. These three important variables had a cumulative contribution of 84.39%. The threshold (presence probability > 0.2) of mean diurnal range (Bio2) ranged from 5.08 to 10.38°C, while mean temperature of the wettest quarter (Bio8) ranged from 3.01 to 25.30°C and precipitation of the driest month (Bio14) ranged from 0 to 48.19 mm (**Figure 8**).

For P_Whole, precipitation of driest month (Bio14, 39.25%) and mean diurnal range (Bio2, 20.99%) made the greatest contributions to the distribution model, and they had a cumulative contribution of 60.24%. The threshold (presence probability > 0.2) of precipitation of the driest month (Bio14) ranged from 0 to 17.61 mm and mean diurnal range (Bio2) ranged from 5.08 to 13.22°C (**Figure 8**).

### Niche Overlap Between Two Populations

**Figure 9:**
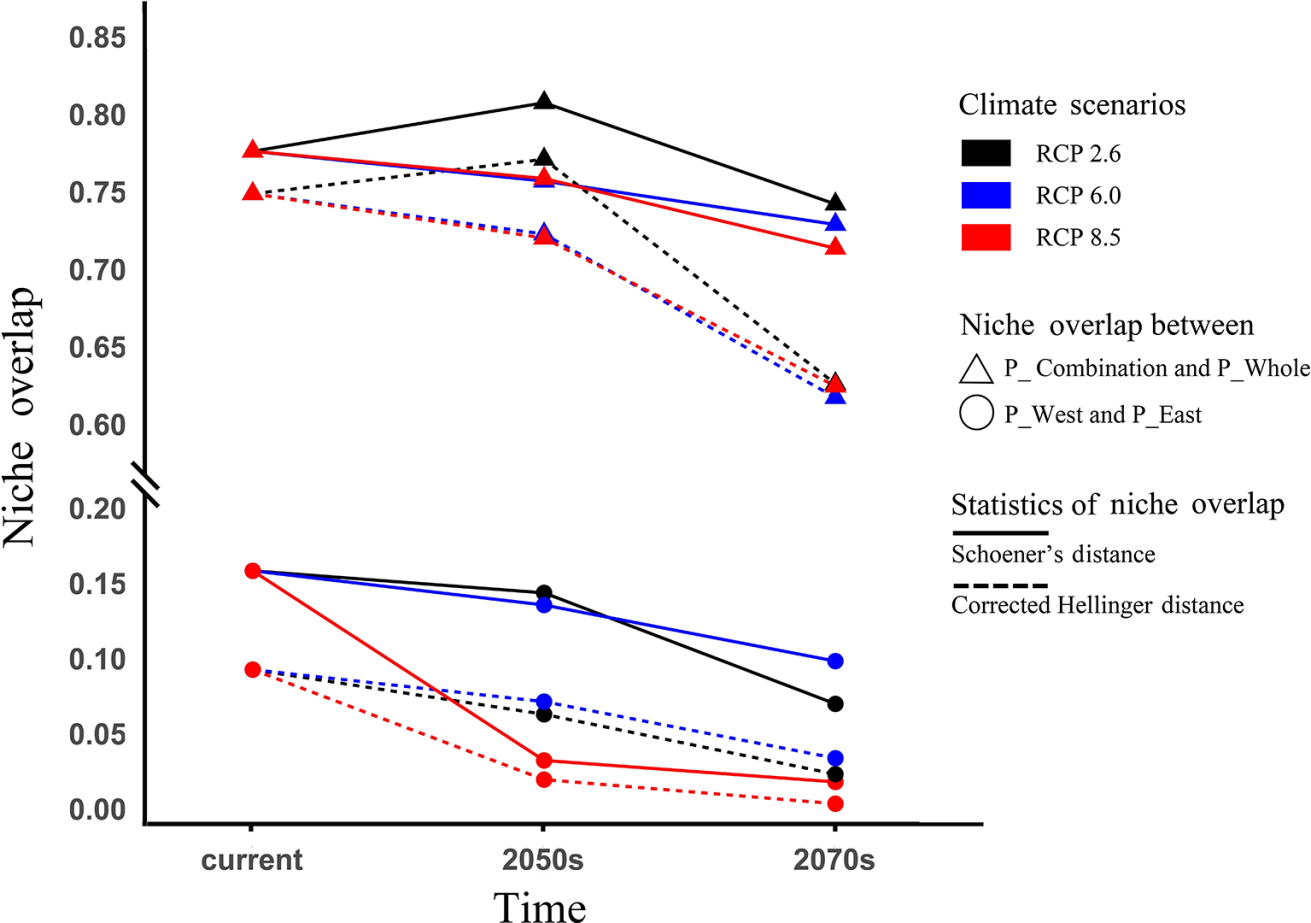
Niche overlap and changes under climate change. Niche overlap was measured by Schoener's D values and corrected modified Hellinger distance, both ranging from 0 (refers to populations with completely discordant niches) to 1 (refers to populations with identical niches).

The current Sch_D and Cor_I between P_West and P_East were 0.09 and 0.16, respectively (**Figure 9**), and 0.77 and 0.80 between P_Combination and P_Whole. Under the three predicted pathways of climate change, they all showed a steadily declining trend over time except for a slight increase that occurred in the 2050s in RCP 2.6. Between P_West and P_East in the last period (2070s), and among the three pathways, Sch_D and Cor_I in RCP 8.5 had the lowest values of 0.004 and 0.018, but the highest values (0.034 and 0.099) in RCP 6.0 (**Figure 9**). Between P_Whole and P_Combination, Sch_D and Cor_I in RCP 2.6 had the highest values of 0.647 and 0.763 in the last period (2070s) among the three pathways.

## Discussion

### Differences Between Populations

Considering that *P. mairei* is an endangered species with spatially distinct populations, we assumed that different populations would possess varying degrees of adaptation to their local environments, and divided all occurrences of *P. mairei* into two populations (P_West and P_East). The results of PCA and HCA partly justified our assumption. The results of PCA suggest that *P. mairei* could be diverging as a result of changes to climate variables such as precipitation and range of annual temperature, while *P. mairei* is thought to be unsuitable under abundant rainfall and adaptive to distinct annual temperature changes (Hong, 2011). Further analysis about climate variables included contributions and response curves of variables in SDMs. Within six chosen climate variables, four variables (Bio02, Bio04, Bio08, and Bio14) had a considerable influence on the distribution of *P. mairei*. Bio2 (mean diurnal temperature range) and Bio4 (temperature seasonality) are both related to temperature changes, while Bio8 (mean temperature of the wettest quarter) and Bio14 (precipitation of the driest month) are related to precipitation and its interaction with temperature.

*P. mairei* is a herbaceous perennial with thick carrot-shaped roots that stores carbohydrates in roots that are crucial for bud development and germination (Walton et al., 2007). In *P. mairei*, a suitable diurnal temperature range might be important for root thickening and further plant development. According to the response curve (**Figure 8**), P_East can survive a much lower diurnal range than P_West, indicating that there is some special local adaption of mean diurnal range for P_East. Mean temperature of the wettest quarter (Bio8) indicates a suitable temperature range for plant growth when there is adequate rainfall, which corresponds to subtropical or temperate monsoon climates in the distribution range of *P. mairei*. Compared to cold, peonies are more sensitive to heat (Hong, 2011), so that the upper limit would contribute more to distribution models. According to the response curve, P_East had a broader temperature range (3.01–25.30°C) than P_West (8.58–22.27°C). *P. mairei* plants respond weakly to flooding stress but are quite strong in response to drought (Hong, 2010), and thus low precipitation in the driest month (Bio14) is needed. On the other hand, excessively insufficient water will affect the normal physiology of seeds, and lead to dormancy, which will made germination difficult (Baskin and Baskin, 2014). According to the response curve, P_West and P_East had the same peak of precipitation (about 10 mm) in the driest month, but P_East already adapted to a higher level of precipitation, the upper threshold (presence probability > 0.2) of P_East being 48.19 mm, compared to 10.17 mm for P_West. While dormancy of peony seeds may result in insufficient water, germination requires a complicated set of temperature conditions: warm stratification for embryo growth and radicle protrusion followed by cold stratification for epicotyl growth (Zhang et al., 2019a). Suitable temperature seasonality (Bio04) is required for the normal germination of *P. mairei*. According to the response curve, the threshold value (presence probability > 0.2) of Bio04 for P_West was 3.33–7.64°C, and 4.7–12.22°C for P_East, indicating that P_East might be more sensitive to temperature and require a higher climate seasonality for seed germination.

The two populations had clearly distinguished geographies, with P_West mostly located in middle Sichuan and P_East mostly located in south Shaanxi. Under the influence of global warming, niche similarity between both populations might decline consistently (**Figure 9**), indicating the strengthened distribution divergence of these populations. Furthermore, as their distributions were predicted to shift in opposite directions, both populations might achieve local adaptation to the new distribution centers, which would strengthen their niche divergence.

### Difference Between Two Approaches

We developed two approaches to construct SDMs for *P. mairei*. The first followed common practice and treated all presence sites within the distribution range as a whole. In the second approach, which assumed that geographically distinct populations would adapt locally to their local environments, separate SDMs were constructed for separate populations. The current distributions of *P. mairei* predicted by these two approaches were basically consistent with each other, and both covered regions where presence sites were located, except that P_Whole lost south Chongqing. When two presence sites existed, south Chongqing was suitable for *P. mairei*. Besides south Chongqing, a larger region was predicted to be suitable for P_Combination than P_Whole, 2.6×10^5^ and 2.4×10^5^ km^2^, respectively. The overlap between P_Combination and P_Whole decreased from the current 0.77–0.64 in the 2070s in RCP 8.5, as measured by Schoener's D value. This finding indicates that the difference between the results from these two approaches arose from climate change, which assigns special consideration to the choice of suitable approaches when preparing to predict the suitable distribution of geographically disjoint populations. Considering that south Chongqing was lost from P_Whole, we suggest that a separate-SDM approach that can correctly evaluate the suitability of a species in regions where presence sites are disproportional, and provide more detail about species distribution, is a more reasonable strategy.

### Conservation Strategies for *P. mairei*

Under the influence of global warming, different changes occurred to the ranges of the two populations. From an overall perspective, most of the current habitats of P_West would be maintained, while those of P_East would become unsuitable in future scenarios.

As we showed, the core distribution of P_West might shift west toward the Tibet Plateau under all RCP scenarios, indicating an upward shift trend for P_West. The elevation gradient of species composition is generally considered to be driven by the corresponding temperature gradient, so species ranges are expected and projected to shift to higher altitudes as climate warms (Lenoir et al., 2008). Indeed, numerous plant species have been reported to move toward higher elevations due to elevated temperatures (Klanderud and Birks, 2003; Lenoir et al., 2008; Wolf et al., 2016; Zhang et al., 2018; He et al., 2019). The areas of suitable distribution of P_West were all predicted to reach a value slightly above the current level by the 2070s under most RCP scenarios (**Figure 6**), which indicates that global warming might benefit P_West. This trend is consistent with other studied peony species, *P. delavayi*, *P. rockii*, and *P. veitchii*, which were modeled to separately expand their highly suitable habitats by 82.35%, 3.00%, and 19.59%, respectively in the 2070s under RCP 8.5 (Zhang et al., 2018; Zhang et al., 2019b). The core distribution of P_East might shift north under different scenarios and tends to include the top of different mountains under different RCP scenarios. The areas of suitable distribution of P_East were all predicted to decline over time under all RCP scenarios, and the decreased values might rise as RCP values rise (**Figure 6**). This suggests that global warming is not likely to be a benefit for the survival of P_East. Both populations showed different responses under different RCP scenarios, indicating the complex mechanism of distribution under different temperatures and/or levels of precipitation. This suggests that more SDMs need to be constructed under different RCPs if there is to be a comprehensive understanding about future trends of distribution changes, which is essential for conservation policy makers.

As our study demonstrates, climatic demand varies within the distribution range of *P. mairei* and might influence its responses to ongoing climate change. If the populations have adapted to these differing conditions, intraspecific variation could be relevant when planning conservation of the species. Experimental studies are needed to disentangle this, but absent such information, readily applicable SDMs may offer one of the best tools to gain insight into the potential importance of niche divergence under climate change. For P_West, implementation of a conservation strategy based on population models could include *in situ* conservation, considering that most current suitable areas were predicted to still be suitable for *P. mairei* under climate change. Thus, efforts should be made to protect these regions from threats produced by anthropogenic activities. Assistance in migration southward is also suggested, considering the long distance from its current distribution to a future climatically suitable distribution in the 2070s, namely in east Yunnan. For P_East, a population-based strategy could entail *in situ* conservation and set core protection areas at highly elevated regions of Qinling and Daba Mountains. In addition, *ex situ* conservation off-site, such as in a botanical garden, is urgent for sustainable research and development of *P. mairei*, as current suitable areas for both populations might shrink or disappear under future climate scenarios. Although SDM is a powerful tool for species conservation, morphological and genetic research is needed to understand intraspecies biodiversity and evolution within *P. mairei*, as these are aspects essential for its effective conservation.

## Conclusion

It is of vital importance to estimate how climate change will affect the distribution of rare species for specific conservation purposes. The results of our study indicate that local climate adaption exists within the distribution range of P. mairei and that different populations respond to climate change quite differently through separate SDMs for separate populations: P_West might shift north, and P_East might shrink from the north, but both are likely to shift upwards driven by rising temperature. By treating all presence sites as a whole would produce a narrower niche, and could miss predicting some suitable areas (south Chongqing in this case). Local adaptation is worth considering in SDM research, and a new approach of constructing separate SDMs for separate populations is suggested. The predicted spatial and temporal pattern of range shifts for P. mairei will be a useful reference for conservation strategies, and the result of population clustering based on PCA and SDMs can be used to help design further genetic research about biodiversity and evolution.

## Data Availability Statement

All datasets generated for this study are included in the article/**Supplementary Material**.

## Author Contributions

XY conceived and designed the experiments. QC, YJY, and RZ performed the experiments. QC and YY analyzed the data. QC and JT drafted the manuscript. JT critically assessed the study. XY and JT revised the manuscript.

## Funding

This work was financially supported by The World-Class Discipline Construction and Characteristic Development Guidance Funds for Beijing Forestry University (2019XKJS0322). This work was also fnancially supported by Forestry Science and Technology Development Project from State Forestry and Grassland Administration (KJZXZZ2019001), P. R. China.

## Conflict of Interest

The authors declare that the research was conducted in the absence of any commercial or financial relationships that could be construed as a potential conflict of interest.
